# Health Disparities: A Perspective on Internal Migration and Health Behavior in Sudan

**DOI:** 10.5334/aogh.2589

**Published:** 2020-05-05

**Authors:** Mahmoud Ali Fadlallah, Indrajit Pal, Joyee S. Chatterjee

**Affiliations:** 1 Disaster Preparedness, Mitigation, and Management, School of Engineering and Technology and School of Environment, Resources and Development, Asian Institute of Technology (AIT), TH; 2 Gender and Development Studies, School of Environment, Resources and Development, Asian Institute of Technology (AIT), TH

## Abstract

**Background:**

Natural hazards, poor socio-economic conditions, low literacy levels, and long-standing conflicts affect traditional gold miners in Sudan and contribute to multiple health vulnerabilities. An extensive survey reveals differential health risk among internal migrant miners leading to short-, medium-, and long-term health consequences and disparities. The need to identify determinants of health behavior and limited prior research on internal migrants involved in traditional gold mining in Sudan motivated this research.

**Objective:**

To investigate potential health disparities between internal migrant workers participating in traditional gold mining and their local counterparts.

**Methods:**

Questionnaires on socio-demographic and health status in the Abideya area in the River Nile state of Sudan were administered to 211 miners. Composite score variables were devised based on existing literature and data for assessment of underlying risk determinants for the miners’ vulnerability (migrants and non-migrants). Six new composite variables were constructed and subjected to analysis by immigration status.

**Findings:**

There are disparities in drivers of health behavior related to the immigration status of traditional gold miners. Access to water, sanitation, and hygiene services are common determinants for the health behavior of both internal migrant miners (p < 0.001) and their local counterparts (p < 0.001). However, knowledge (p < 0.05) and perception (p < 0.05) are additional critical determinants for the health behavior of local miners, while education (secondary, p < 0.01) is an additional modifier for the immigrants’ health behavior.

**Conclusions:**

The outcomes of this field-based research suggest increased awareness and risk perceptions among migrants could improve health-related behaviors. The study advocates for policymaking and implementation of health programs at all levels to reduce health disparities between migrants and non-migrants, improving the health status of the entire community.

## 1. Introduction

Migration is defined as a process of moving across international borders or within a country. It encompasses any kind of movement of people, whatever its length, composition, and motivation [1]. It is a critical process of social and demographic change. Globally, migrant workers represent about 72.7 percent of the 206.6 million working-age migrants (15 years of age and over), with the majority (83.7 million) men [2]. People who move internally may continue to another country, most likely to a neighboring country, and then to another continent [3]. Both internal and international migration are common, and both have short- and long-term implications for migrating individuals and for communities.

Internal migration is defined as “Movement of people from one area of a country to another area of the same country for the purpose or with the effect of establishing a new residence”. This migration may be temporary or permanent. Internal migrants move but remain within their country of origin [1]. The definition, by default, includes internally displaced persons (IDPs) who have been forced or obliged to flee their homes in response to stressors. Therefore, internal migration is a consequence of the unequal distribution of resources, opportunities, and services or a result of violence and natural or human-made disasters [4].

Health research related to migrants tends to put more emphasis on various aspects related to international rather than internal migrants. Limited but important studies within the internal migrant domain have shed light on aspects related to the “healthy migrant effect”, in which healthier individuals are more likely to migrate and to travel further from home [5,6], and the “salmon bias” phenomenon, in which migrants return to their places of origin when their health status deteriorates or they face health challenges [6,7], Despite these important phenomena, internal migrant-related health disparities have not yet been the subject of significant research focus.

The process of migration and the health of migrants are connected in complex ways. The concept of migration and health encompasses the idea that there are various factors and conditions that influence the health of migrants and make them vulnerable to poor health outcomes [8,9]. Further, migration has been found to be one of the social determinants of health that contributes to aggravate health disparities between migrants and local communities [3,10,11]. For instance, studies indicate that there may be differences in health risk factors and disease profiles between migrants and local populations, or inequalities in the access or utilization of preventive services and in health outcomes [12,13,14].

Many studies portray migrants as being in poor health and a burden on health systems [15]. Migrants frequently face disadvantages during migration, such as poor living conditions, discrimination, stigma, inequity, and poor social and community support in the host destination, which can negatively impact their health over time [3,14]. Apart from the social challenges of migration, migrants face health challenges concentrated around the issues of healthcare accessibility, affordability, entitlement, responsiveness, acceptability, quality, and inequity [3,8,16]. Previous studies have shown that documentation status, lack of health insurance, language and communication challenges, socioeconomic status, and gender are the most common barriers preventing migrant laborers from accessing healthcare [14,17,18].

Health status due to work-related differences among the migrant population has been studied in many parts of the world. In European countries, for instance, migrant workers were more likely found to be exposed to unhealthy working conditions and employment arrangements—high temperature, loud noises, vibrations, fast work speed, and standing for a longer time—than the native workers [19]. Similarly, in Canada, a research study compared differences between migrant workers and their Canadian-born counterparts concluded that most migrant workers were involved in unsafe working conditions (e.g. working longer hours, performing physically demanding jobs) and were persistently exposed to hazards without sufficient workplace protections [20]. Migrant workers were found to experience increased vulnerability due to limited knowledge about occupational health and safety and limited participation in risk prevention due to lack of voices and participation in social processes. Furthermore, in the Czech Republic, Sweden, United Kingdom, and Spain work-related injuries and fatal accidents were more common among migrant laborers than non-migrant populations [21,22,23]. This signifies that migrant workers are potentially exposed to differing levels of health risks and experience unique vulnerabilities in workplaces (and communities), as evidenced through existing health disparities when comparing migrant and non-migrant populations in the same workplace.

In light of the gaps in previous research on migrants, the current paper specifically examines factors affecting internal migration and health in the context of the traditional gold mining industry in Sudan.

## 2. Internal Migrants in Sudan and Traditional Gold Mining

Sudan, due to its strategic geographical location, is a source, transit, and destination country for international migrants. According to the International Organization for Migration (IOM), migrants in Sudan are from 17 different countries of origin [24], with Eritrea, Ethiopia, Nigeria, Somalia, and Syria representing the top five countries of origin. However, for the majority of migrants, Sudan acts as a corridor through which they proceed to countries such as Canada, the USA, the United Kingdom, Australia, the Kingdom of Saudi Arabia, and some European countries such as Germany, Sweden, Norway, and Switzerland. International organizations such as the IOM are active in assessing the status of international migrants.

Internal migration in Sudan has occurred since the 1970s, when the people started to flee rural areas as a result of conflicts and natural disasters such as droughts, along with inequitable distribution of resources. This rural-urban migration intensified urban poverty as more people became unemployed, lacked access to social services, and lived in informal settlements around the urban areas [25]. Between 1983 and 2005, a large number of people who already lived as internally displaced persons (IDPs) moved to urban areas to gain access to social services and economic opportunities [26]. Those people became vulnerable to multiple threats and challenges affecting their physical and mental wellbeing, and the move also resulted in poor or limited access to livelihoods, basic services and land [26]. Additional pressure was also imposed on local services and goods, and increased incidents of robberies, theft, and begging.

As gold mining opportunities exist mostly across rural areas, the recent gold rush in Sudan has intensified and stimulated internal migration (rural-rural, urban-rural) countrywide, and ultimately attracted a large number of migrant workers. As of 2015, government statistics estimated that more than one million miners are directly employed, spread over 14 of the 18 states of Sudan [27]. This figure does not distinguish between local and migrant miners.

Miners, like other migrant workers, are considered to be motivated by the livelihood income from the mining operations [28,29]. They initially seek employment as temporary workers to collect a certain amount of wealth, then travel back to their place of origin or elsewhere to start a new business(s) or to expand existing ones. Some leave the mines when they become sick, witness accidents, lose their jobs, or otherwise experience economic downturn [16,28]; some primarily work in the mines on a seasonal basis [30]. Conversely, some miners continue to work in the mines for longer periods due to the opportunities to grow their income or because they have no alternative sources of income [31].

Despite the economic benefits of gold mining to individuals and countries, the health challenges are more severe and put the miners at high risk of future health impacts. Miners are persistently exposed to environmental and occupational health hazards [32,33,34] and lack access to quality healthcare and social services [35]. They are usually engaged in dangerous manual labor and frequently utilize rudimentary or unsafe mining and mineral processing methods. Besides, poor environmental health conditions are created as a result of water shortages, consumption of unsafe (contaminated) water, poor sanitation, food hygiene, and waste disposal practices [31]. Moreover, a lack of knowledge and technical training, poor access to information about hazards, and a low level of perceived risk fuel unsafe health behaviors among members of mining communities [36,29].

As it has been shown that other types of migrant workers—whether in-country or cross-border migrants—are more vulnerable and exposed to higher levels of health risks relative to their local peers [13,14,37], migrant miners are expected to have a similar situation. Unfortunately, the policies and legislation in Sudan on migration do not specifically consider health issues related to migration. Instead, they address affairs broadly related to Sudanese labor migration, irregular migration, employment of foreigners, refugees, asylum seekers, and human trafficking [25].

Faced with these gaps, the current paper seeks to advance knowledge regarding the health of traditional gold miners and aims to answer several questions: Are there potential health disparities between internal migrant workers and their local counterparts? Do the determinants of health behavior, one type of health disparity, differ by immigration status? To the best of our knowledge, no similar or relevant studies have dealt with these matters in Sudan.

## 3. Materials and Methods

### 3.1. Study area

**Figure 1:**
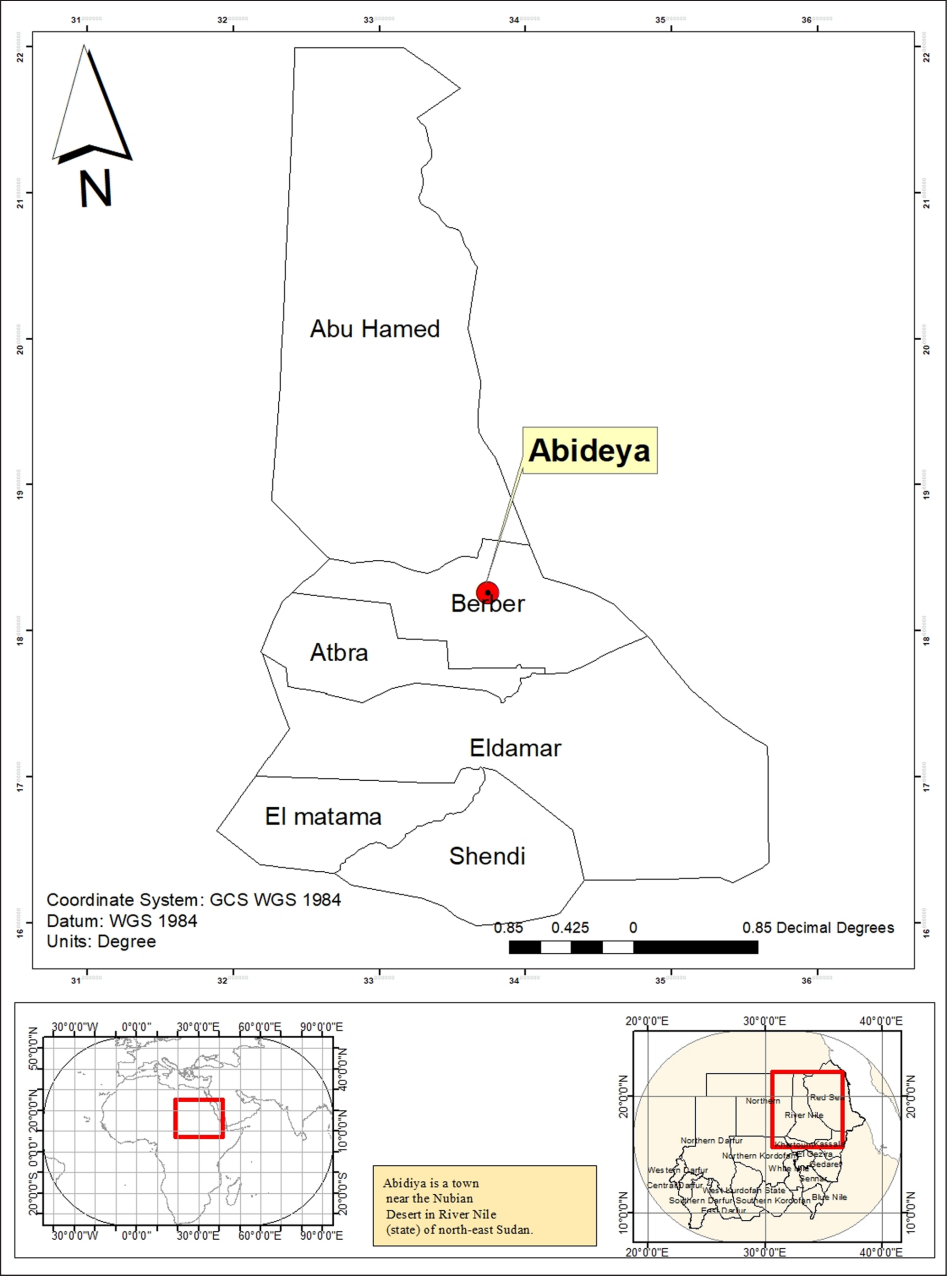
Location of the study area, Abideya, Berber locality of the River Nile state.

The study was conducted in Abideya in the Berber locality of the River Nile state of Sudan (Figure 1). This area hosts major gold mining operations as well as the largest market in the country. Reports from the government have estimated that River Nile state accounts for more than 60% of the total gold produced in the country (Abideya Administrative Unit, March 2018, Unpublished report). Almost 85% of this gold is produced by traditional mining processes [27].

### 3.2. Sampling

Since traditional gold mining processes are carried out in two types of locations, mine sites and processing centers, the sampling procedure adopted in this study followed a quota approach. In this approach, miners were stratified before the field survey into two distinct groups. The two groups included those who work in nearby mine sites and those who work in processing centers in the large Abideya gold market. Respondents were approached based on their availability and consent during fieldwork. The inclusion criteria are traditional gold miners, aged 18 or older, who have worked for at least one year in the area of Abideya.

We followed Cochran’s procedure of sample size calculation [38] (Equation 1), with an initial sample size of 196 respondents with a 95% confidence interval and 7% precision level. An additional 7.5% was considered to cater to non-respondents or missing values to increase the accuracy of the results. Eventually, a total sample of 211 respondents were interviewed during the current study. The total number of respondents was further redistributed based on their status (migrant and non-migrant).

In this study, mineworkers from outside Abideya were considered migrants in the operational sense. Indeed, non-local miners from different regions across Sudan have been classified as a uniform migrant category. The final sample redistribution classified 63 (about 30%) respondents as local, and 148 (about 70%) as migrant respondents.

Equation 1 {n_0} = \frac{{{Z^2}pq}}{{{e^2}}}

Where:

n_0_ = Sample size

Z = Confidence level (±1.96 at 95%)

p = the estimated proportion of an attribute that presents in the population (0.5)

q = 1 – p

e = The desired level of precision, (0.07 = ±7)

Thus,

Initial sample size required = [(1.96)^2^ × (0.5) × (1–0.5)]/(0.07)^2^ = 196

Final sample size calculated = 196 + (196 × 0.075) = 210.7 ≈ **211**

### 3.3. Data collection method

Data were collected from January to February 2018 using an interviewer-administered questionnaire and survey conducted by the authors and public health officers trained for this purpose.

The questionnaire was structured into three sections. The first section includes socio-demography, health status, and access to healthcare as background variables. The second section includes healthcare-seeking behavior, risk perception, and knowledge-related variables. The third section includes health behavior and practice variables. A pilot group of 10 miners was given the questionnaire before survey implementation to test clarity and level of understanding of questions, which were improved accordingly.

### 3.4. Variable selection and analysis

Various demographic, socioeconomic, health and environmental health variables were included to ensure a multi-dimensional approach toward understanding disparities. A rigorous selection procedure was applied for variable selection, using a clustering technique to group the variables closely explaining a certain phenomenon. The resultant cluster was given the name of such a phenomenon. Iterative processes for variable transformations were carried out to form sets of variables having similar scales of measurement under each designated cluster (Table 1).

**Table 1:** Variables/indicators selected to study health-related disparities.

Cluster	Variable/Indicator	Expression/Coding	Measurement

	Age in years		Discrete
	Years of work		Discrete
	Income/month Sudanese Pound (SDG)		Discrete
	Education	Illiterate (1)	Nominal
		Informal (2)	
		Primary (3)	
		Secondary (4)	
		University (5)	
Mental health	Experiencing stress	No (0)	Nominal
		Yes (1)	
Physical health	Having chronic disease	No (0)	Nominal
		Yes (1)	
Access to healthcare	-Distance to the nearest health facility	>5 km (0)	Nominal
		<5 km (1)	
	-Means of transportation to a health facility	On foot/Public transport (0)	Nominal
		Taxi/renting a car/Own vehicle (1)	
Healthcare-seeking behavior	-Medical care received in the previous six months	No (0)	Nominal
		Yes (1)	
	-Health care sought in a health facility	No (0)	Nominal
		Yes (1)	
	-Health care sought in the nearest health facility	No (0)	Nominal
		Yes (1)	
Access to water, sanitation, and hygiene (WASH) services	-Drinking water source	Well/Surface water/Very poor (1)	Likert scale
		Tanker truck/Poor (2)	
		Water Tank/Moderate (3)	
		Piped/Bottled water/Good (4)	
	-Sanitation/latrine	Bucket/Bush/Field/Very poor (1)	Likert scale
		Pit without slab/Poor (2)	
		Pit with slab/Moderate (3)	
		Flush to septic/Good (4)	
	-Shower location	Open area/Very poor (1)	Likert scale
		Inside tent/dwelling/Poor (2)	
		Inside toilet/Moderate (3)	
		Inside private shower/Good (4)	
Perception	-Perception of mining-related risks	Very low (1)	Likert scale
		Low (2)	
		Moderate (3)	
		High (4)	
		Very high (5)	
	-Self-rated nutritional status	Very low (1)	Likert Scale
		Low (2)	
		Moderate (3)	
		High (4)	
		Very high (5)	
	-Perceived adherence to safety measures	Very low (1)	Likert scale
		Low (2)	
		Moderate (3)	
		High (4)	
		Very high (5)	
	-Perceived quality of healthcare services	Very low (1)	Likert scale
		Low (2)	
		Moderate (3)	
		High (4)	
		Very high (5)	
	-Perceived satisfaction regarding accessing necessities	Very low (1)	Likert scale
		Low (2)	
		Moderate (3)	
		High (4)	
		Very high (5)	
Knowledge	-Health hazards		Discrete
	- Traditional gold mining-associated diseases/health outcomes		Discrete
	- Methods of diarrhea prevention		Discrete
Health behavior	-Water treatment practices	No (0)	Nominal
		Yes (1)	
	-Garbage disposal practices	No/Throw it outside (0)	Nominal
		Yes/In a public bin/In a bin inside/In a sack or carton outside (1)	
	-Shower habits (personal hygiene)	No/Every two days or more (0)	Nominal
		Yes/Every day/Every other day (1)	
	-Handwashing habits(personal hygiene)	Inadequate/Twice or less (0)	Nominal
		Adequate/Three times or more (1)	
	-Having health insurance	No (0)	Nominal
		Yes (1)	

Before developing composite scores using the average score [39], factor analysis using Principal Component Analysis (PCA) with varimax rotation was used in this study to extract the underlying construct(s) and test whether the selected variables might hold together in explaining a certain phenomenon, i.e., to separately test the construct validity of each set of grouped variables. PCA has been used in academic research to devise indexed or composite variables [40,41]. Each PCA test giving a result with a Kaiser–Meyer–Olkin (KMO) measure of sampling adequacy ≥0.50 and a statistically significant Bartlett’s test of sphericity (p < 0.05) was kept and considered in the analysis.

However, knowledge variables with different scales of measurement were discrete in nature because of the variable response in terms of subjective and objective health hazards and health outcomes potentially associated with traditional gold mining activities. The more responses that were given to each variable, higher the score recorded for the respondent. Then, following the PCA method to test the construct validity of variables, standardized values were obtained using the Z-score method [42] (simple averaging) to develop a composite score for knowledge variables.

The entire dataset for this study was analyzed using SPSS (SPSS Statistics for Windows v. 22 IBM, USA). The newly constructed composite variables, as well as other discrete variables, were then tested against immigration status using the Mann-Whitney U test, while the other categorical variables were tested for differences using Chi-Square values. Data were also split by immigration status to run a separate regression analysis for each group.

## 4. Profile of Respondents

A total of 211 male traditional gold miners enrolled in the study. Of these respondents, 115 (54.5%) represented mining sites, and 96 (45.5%) represented processing centers. The average age of the local miners was 28.76 years (standard deviation (SD) = 8.17), the minimum was 1 year and the maximum was 60 years (Table 2). Similarly, the average age of the migrant miners was 30.18 years (SD = 9.32), and the range was between 1 to 61 years. There was no statistically significant difference observed in age distribution across local and migrant workers (p > 0.05). The median years of work for both groups of miners were 4.0 years. It can be seen that, on average, migrant workers tend to stay and work more years (5.8 years) compared to local workers (4.8 years) and the difference was statistically significant (p < 0.05). The average monthly income of local miners was 5,142.86 Sudanese Pounds (SDG) (SD 2,517.34), with a minimum of 1,000 SDG and a maximum of 15,000 SDG. Similarly, the average monthly income of migrant miners was 5,341.22 (SD 2417.36) with a minimum income of 2,000 and a maximum income of 15,000 SDG. There was no statistically significant difference observed in income across both groups. Although the majority of respondents were married, there was no significant difference in the marital status between migrant and non-migrant miners (p > 0.05). About one-third of the respondents (34%) were university graduates and 16% had no education. Notably, a little more than half of the migrants were illiterate or having just primary education. This means that migrant miners have attained less education compared to their local counterparts (p < 0.01). Based on their geographical origin, one-third of the respondents were local miners, one third migrated from the western part of the country where protracted conflicts and poverty are predominant, and the remaining miners surveyed were from various regions across the country. Out of the total sample, only 3.4% reported having health insurance, indicating poor access to social services. Indeed, migrant respondents were the most vulnerable where 99% were out of health insurance coverage compared to 90% of local respondents (p < 0.01). As of 2017, the social health insurance coverage reached about 54% of the population of Sudan [43]. Of the people covered, only about 22.5% were workers in the informal sector, which includes traditional gold miners. The uninsured traditional gold miners pay out-of-pocket to receive healthcare services.

**Table 2:** Respondents’ sociodemographic characteristics, N = 211 (Local: 63, Migrant: 148).

Characteristic	Local			Immigrant			Test of difference	Sig.

	Min-Max	Median	Mean (SD)	Min-Max	Median	Mean (SD)		

Age (years)	18–60	27	28.76 (8.17)	18–61	29.0	30.18 (9.32)	Mann-Whitney U test	0.271
Years of work	1.0–20.0	4.0	4.89 (3.67)	1.0–20	4.0	5.83 (3.75)	Mann-Whitney U test	0.034*
Monthly income (SDG)	1,000–15,000	5,000	5142.86 (2517.34)	2,000–15,000	5,000	5341.22 (2417.36)	Mann-Whitney U test	0.703
Marital status:	**Freq. (%)**			**Freq. (%)**				
Unmarried	31 (49.2)			59 (39.9)			Pearson Chi-Square	0.209
Married	32 (50.8)			89 (60.1)				
Education:								
Illiterate	7 (11.1)			25 (16.9)			Pearson Chi-Square	0.001**
Informal	0 (0.0)			1 (0.7)				
Primary	7 (11.1)			50 (33.8)				
Secondary	16 (25.4)			33 (22.3)				
University	33 (52.4)			39 (26.3)				
Having health insurance								
No	57 (90.5)			147 (99.3)			Pearson Chi-Square	0.001**
Yes	6 (9.5)			1 (0.7)				

* Denotes significance at 0.05.

## 5. Results

### 5.1. Principal component analysis (PCA) method

As all composite variables showed highly significant Bartlett’s test of sphericity (p = 0.000) and a quite significant variance explained (Table 3), we decided to keep the composite variables even though the KMO was less than 0.60. Low KMO values might be due to a smaller number of variables used under each cluster.

**Table 3:** Results of principal component analysis with varimax rotation.

Cluster	Kaiser-Meyer-Olkin	Bartlett’s Test Significance	Total Variance Explained (%)

Access to healthcare	0.50	0.000*	67.778
Healthcare-seeking behavior	0.52	0.000*	50.310
Access to WASH services	0.50	0.000*	77.652
Perception	0.50	0.000*	52.127
Knowledge/awareness	0.53	0.000*	60.342
Health behavior	0.56	0.000*	62.856

* P < 0.001.

Three items were eliminated from the analysis (having accident experience, having symptoms of mercury poisoning, willingness to pay for health insurance) because they failed to meet the minimum criterion of having a primary factor loading of ≥0.4 in their respective clusters. As a result, six new composite variables were constructed: access to healthcare; healthcare-seeking behavior; access to water, sanitation, and hygiene (WASH); perception of risk; knowledge/awareness of health hazards, health outcomes, and methods of diarrhea prevention; and health behavior (Table 3). Furthermore, an additional six variables, not subjected to PCA, were considered separately in the analysis: stress, chronic disease status, age, years of work, education, and income.

Table 3 shows that the cluster of access to WASH services has the highest variance explained (77.65%), followed by the clusters of access to healthcare and health behavior, which explained 67.78% and 62.86% of the variance, respectively. The smallest amount of variance explained (50.31%), belongs to the healthcare-seeking behavior cluster.

### 5.2. Measuring difference by immigration status

Of the total respondents surveyed, 30% were local and 70% migrant miners. In addition to six individual variables, each newly formulated composite variable was subjected to a test of difference by immigration status. The results confirmed that differences in education, reported stress, healthcare-seeking behavior, knowledge related to health hazards, health outcomes, and methods of diarrhea prevention, and health behaviors/practices were statistically significant (p < 0.05) between internal migrants and local people (Table 4).

**Table 4:** Tests of difference by immigration status across composite and individual variables.

Variable	Test	P-value

Age	Mann-Whitney U test	0.271
Years of work	Mann-Whitney U test	0.034*
Income	Mann-Whitney U test	0.703
Education	Chi-Square (18.765)	0.001*
Experiencing stress	Chi-Square (4.104)	0.043*
Having chronic disease	Chi-Square (0.036)	0.850
Access to healthcare	Mann-Whitney U test	0.927
Healthcare-seeking behavior	Mann-Whitney U test	0.001*
Access to WASH	Mann-Whitney U test	0.755
Perception of risk	Mann-Whitney U test	0.140
Knowledge related to health hazards, health outcomes, and methods of diarrhea prevention	Mann-Whitney U test	0.001*
Health behavior	Mann-Whitney U test	0.050*

* The significance level is 0.05.

In Table 3, it can be seen that five variables are statistically significant based on immigration status. Three variables (education, Knowledge related to health hazards, health outcomes, and methods of diarrhea prevention, healthcare-seeking behavior) showed strong statistical significance (p < 0.01). Two variables, health behavior and reported stress, exhibited relatively lower statistical significance (p ≤ 0.05) compared to other statistically significant variables.

Fifty-four respondents reported having experienced stress. Of these, 59% were immigrants and 41% were non-immigrant miners. The results revealed a statistically significant difference between the two groups (p < 0.05).

Out of six respondents who reported having chronic diseases, four respondents were immigrants. However, no statistically significant variation was observed between immigrant and non-immigrant miners.

In terms of access to healthcare, about 47% of immigrants and the same percent of non-immigrants were not covered and reported having access to services within more than five-kilometer radius which is larger than the standard geographical distance of coverage, within five kilometers. The majority of immigrants (73%) and non-immigrants (75%) relied on using either taxi or their private vehicles to get to healthcare facilities. In both cases, immigrants and non-immigrants have no difference in access to healthcare services (p > 0.05) but it’s indicative of a common vulnerability related to access to healthcare services.

Interestingly, healthcare-seeking behavior showed a strong statistically significant difference among the groups of miners. Using the mean values of the score, local miners were better off and having higher values related to healthcare-seeking behavior (0.714, SD 0.310) compared to their local migrant counterparts (0.574, SD 0.288).

Access to WASH services showed no statistically significant difference between immigrant and non-immigrant miners. However, among all immigrants, only about one third scored more than the average value hence having improved access to WASH services. Comparatively, about 38.0% of local miners scored more than the average value and therefore have improved access. In both cases, whether immigrants or non-immigrants, more than 60% of miners had poor access and scored below the average value.

Regarding the perception of risk, the results showed no difference between the immigrant and non-immigrant miners. However, 57% of non-immigrant miners scored above the average value and thus had a high level of risk perception compared to only 44% of immigrants who scored above the average value.

Only about 41% of immigrants scored above the average value of knowledge variable compared to about 50% of non-immigrants who scored above the average value. Interestingly, this difference was shown to be highly statistically significant.

About 44% of immigrants scored below the average value of health behavior variable compared to only about 30% of non-immigrants who scored below the average value. This shows that the health behavior of non-immigrants was likely to be better than that of immigrants. This difference was just as statistically significant.

### 5.3. Stepwise multiple linear regression analysis

A final test was conducted using stepwise multiple regression analysis (linear regression through the origin), considering the composite variable health behavior as the dependent variable. A test of normality was conducted for the dependent variable and five cases (outliers/extreme values) were replaced by the average value. Further, education, as a categorical variable with more than two categories, was transformed into a dummy variable before regression. All composite variables and individual variables were included in two models segregated by immigration status, i.e. one model for migrant cases and another for non-migrant cases. This procedure allowed the separate relation of statistically significant variables to immigrants and non-immigrants.

The final model resulting from the stepwise regression for immigrant cases (model two), is quite satisfactory. It is statistically significant (ANOVA F = 321.180, p = 0.000) and explains 81.2% (adjusted R^2^) of the variance in the dependent variable (Table 5a). The statistically significant variables are access to WASH (p = 0.000) and education (secondary) (p = 0.002) (Table 5b). These two variables resulted in positive coefficients, indicating a direct relationship with the health behavior of immigrant miners.

**Table 5:** Stepwise multiple linear regression for immigrants.

a) Model summary					
Model	R		R^2^	Adjusted R^2^	Std. Error of the Estimate

	Immigration status = Immigrant (Selected)	Immigration status ~ = Immigrant (Unselected)			

1	0.895		0.802	0.801	0.16469
2	0.903	1.000	0.815	0.812	0.15976

**Table:** 

b) Coefficients								
**Model**		**Unstandardized Coefficients**		**Standardized Coefficients**	**t**	**Sig. (P-value)**	**95% ConfidenceInterval for B**	
	
		B	Std. Error	Beta			Lower Bound	Upper Bound

1	Access to WASH	0.123	0.005	0.895	24.390	0.000	0.113	0.133
2	-Access to WASH	0.115	0.005	0.838	21.017	0.000	0.104	0.126
	-Secondary	0.100	0.031	0.127	3.195	0.002	0.038	0.161

Similarly, the final model resulting from stepwise regression for non-immigrant cases (model three) is statistically significant (ANOVA F = 162.950, p = 0.000) and explains 88.5% (adjusted R^2^) of the variance in the dependent variable (Table 6a). Variables that showed significance are access to WASH (p = 0.000), knowledge of health hazards, health outcomes, and methods of diarrhea prevention (p = 0.025), and perception of risk (p = 0.036) (Table 6b). All significant variables had positive coefficients and thus were directly related to the health behavior of non-immigrants (Table 6b).

**Table 6:** Stepwise multiple linear regression for non-immigrants.

a) Model summary					
Model	R		R^2^	Adjusted R^2^	Std. Error of the Estimate

	Immigration status = non-immigrant (Selected)	Immigration status ~ = non-immigrant (Unselected)			

1	0.931		0.866	0.864	0.14341
2	0.939		0.882	0.878	0.13562
3	0.944	1.000	0.891	0.885	0.13177

**Table:** 

b) Coefficients								
**Model**		**Unstandardized Coefficients**		**Standardized Coefficients**	**t**	**Sig.(P -value)**	**95% Confidence Interval for B**	
	
		B	Std. Error	Beta			Lower Bound	Upper Bound

1	Access to WASH	0.132	0.007	0.931	20.034	0.000	0.119	0.146
2	-Access to WASH	0.127	0.007	0.893	19.485	0.000	0.114	0.140
	-Knowledge	0.066	0.023	0.132	2.886	0.005	0.020	0.112
3	-Access to WASH	0.082	0.022	0.575	3.728	0.000	0.038	0.126
	-Knowledge	0.053	0.023	0.106	2.300	0.025	0.007	0.099
	-Perception	0.041	0.019	0.339	2.148	0.036	0.003	0.080

## 6. Discussion

The limited body of research addressing health disparities among internal migrants working in the traditional gold mining sector motivated this study’s attempt to examine disparities between migrants (in-country) and non-migrants (their local counterparts). A further step comprised an attempt to recognize the determinants of health behavior, an important factor contributing to health disparities and representing a major concern for traditional gold miners [31,36].

The main findings of the first stage of the analysis showed that there are statistically significant health disparities by immigration status. The contributing factors include education, reported stress, healthcare-seeking behavior, knowledge of health hazards, health outcomes, and methods of diarrhea prevention, and health behavior (Table 4). Since this stage of analysis highlighted the existence of health disparities, a second stage of the analysis that utilized the health behavior composite variable as an outcome variable in the regression model was implemented to study its drivers by immigration status.

Looking at the significant variables with substantial influence on the health behavior of migrant miners, it appears that access to WASH services is an important determinant (Table 5b). Generally, 63.5% and 28.2% of rural households in Sudan have improved access to the drinking water source and sanitation facilities, respectively. When combining both, the general situation is even worse where only 19.1% of rural households have access to improved water and sanitation [44]. Therefore, it’s not surprising to observe this factor having a significant influence on health behavior among both migrants and non-migrants.

Environmental health services and consequent outcomes [45] are areas of great concern for all miners since limited facilities and services of poor quality (if any exist) are all that is available to them [31], a situation resembling that reported in Indonesia [46]. Miners in Indonesia have limited access to safe water and defecation or toilet facilities. Instead, they rely on the surface water as their main source of drinking water and use the forest, river, or beach for defecation. These behaviors may contribute to the spread of contagious diseases.

The second important determinant for the health behavior of migrant miners is education (Table 5b). In this regard, as migrants’ educational level increases, their health behavior also improves. Indeed, migrants display a poor education status compared to their local counterparts; a significant proportion (about 51%) reported no or only a primary-school level of education. The main implication for migrants is that access to information on hazards and risks might be mediated by their education level, and their health behavior changes accordingly. In contrast, non-migrants’ educational level showed no influence on their health behavior. Additional factors, such as knowledge of health hazards and health outcomes and risk perception, influenced non-migrant miners’ health behavior (Table 6b).

Regarding risk perception, local miners are more familiar with and are more exposed over a longer period to risks associated with mining operations taking place in the area. Thus, their perception of risk could be higher, and contribute much more to influencing their health behaviors compared to migrant miners. This result differs from the conclusions of Yong et al., who stated that, in the context of risk communication and management in natural disasters, levels of risk perception were similar among immigrants and locally-born individuals [10].

In this study, migrant miners’ perception of risk was found to play no role in modifying their health behavior. No matter the risk, migrant miners, who often come from economically deprived regions, seem to focus on the potential income from gold production [28,46]. Therefore, migrant miners might have a higher level of acceptable risk (risk tolerance) compared to their local peers.

Moreover, knowledge (awareness) related to health hazards, health outcomes, and methods of diarrhea prevention of non-migrants appears to be a critical determinant influencing their health behaviors. Local people are better aware of and familiar with their environment and related hazards and risks due to the easy access to and sharing of information through the customary social support networks [47]. This might support the idea that local people possess a better perception of risks. Migrant miners’ knowledge seems to play no role in driving health behavior, which in turn could affect aspects of occupational health and safety [20]. This might be due to factors related to education, access to information, and the tendency to stay with the same migrant groups (often relatives or others from the same region), leading them to be less socially integrated with local people.

The background variables of age and income showed no evidence of significance by immigration status. Further, the existence or lack of stress and/or chronic disease showed no influence on the health behavior of both local and migrant miners. Additionally, access to healthcare and healthcare-seeking behavior did not contribute to modifying the health behavior of either group of miners. This might be due to the common, harsh conditions that the migrant and non-migrant miners face, including a lack of access to healthcare and social service systems.

## 7. Conclusion

Recent recommendations from the global conference (Global Consultation on Migration and Health) held in Colombo, Sri Lanka in 2017, stated that gathering evidence concerning migration and health will necessitate an understanding of the nexus between migration and health and exploration of health issues across different classes of migrants [8]. Similarly, Chung & Griffiths (2018) recommended that interdisciplinary work is required to improve the aspects of health status, health care delivery, and related public policy in the migration domain [11].

The evidence from the present study suggests that the nexus between migrants and health can also be fruitfully explored through the experiences of internal migrant workers in traditional gold mining.

Although internal migrants working as traditional gold miners experience increased health risks in Sudan, the government and healthcare providers have only a limited awareness of their needs due to the lack of clear policies related to migration and health, along with the existence of competing priorities, such as communicable diseases.

In the present study, health-related differences were observed between migrant and non-migrant miners. Traditional gold miners are exposed to high levels of health hazards and risks associated with mining operations [31,32,33,34]. Drivers of health behavior were found to differ by immigration status, except for access to WASH services, which is common to both strata.

Education is an area of concern for migrants, while knowledge (awareness) related to health hazards, health outcomes, and methods of diarrhea prevention, and perception of risk are areas of concerns for their local counterparts. Interventions should seek to raise the awareness and risk perception of internal migrant miners to improve their health behavior, and ultimately, health outcomes. Future research should focus on examining the differences in health outcomes and tracking the effect of “salmon bias” among internal migrant miners. Additionally, exploring the role of social relations and support in determining the health behavior of miners segregated by within-country immigration status is a promising area for future research.
